# Supplementary material for: Modelling transmission and control of the COVID-19 pandemic in Australia
Source: Nat Commun. 2020 Nov 11;11:5710. doi: 10.1038/s41467-020-19393-6 (PMC7659014; doi:10.1038/s41467-020-19393-6)
Supplement: Supplementary file 1 — Supplementary Information [file 41467_2020_19393_MOESM1_ESM.pdf]

Modelling transmission and control of the COVID-19 pandemic in Australia Supplementary Information

Sheryl L. Chang[1], Nathan Harding[1], Cameron Zachreson[1], Oliver M. Cliff[1], and Mikhail Prokopenko[1,2,*]

[1] *Centre for Complex Systems, Faculty of Engineering, University of Sydney, Sydney, NSW 2006, Australia*
[2] *Marie Bashir Institute for Infectious Diseases and Biosecurity, University of Sydney, Westmead, NSW 2145, Australia*
[*] *Corresponding author: mikhail.prokopenko@sydney.edu.au (ORCID: 0000-0002-4215-0344)*

A. COVID-19 pandemic in top 8 affected countries and Australia

As of 21 March 2020, when significant intervention measures were introduced in Australia, over 285,000 cases have been confirmed worldwide, causing more than 11,500 deaths; and in a month, by 23 April, the total number has grown to exceed 2.628 million cases, with more than 183,400 deaths [1, 2]. By 21 March 2020, the disease established a sustained local transmission in many countries around the globe, with more than 180 countries and territories affected, including Italy, Spain, Iran, the United States, Germany, France, and South Korea as the top eight affected nations [1, 2].

The scale of the COVID-19 pandemic has grown several orders of magnitude in a matter of weeks, from hundreds to thousands to tens of thousands, with the rate of these transitions varying across countries. Of particular interest to our study is the time periods when the epidemics are sustained locally in these countries, but before the effects of adopted intervention strategies are fully felt. One immediate observation is that during this period, the growth rate of cumulative incidence in many of the traced national epidemics is averaging within the range between 0.2 and 0.3 per day, that is, there are 20% to 30% daily increases in new cases on average. This is particularly evident for Spain, France, and Germany (Supplementary Fig. 2), as well as China, Iran and Italy (Supplementary Fig. 1). These average estimates provide approximate "invariants" and reduce uncertainty around key epidemiological parameters, required to calibrate disease transmission models, before investigating possible effects of various intervention policies.

Supplementary Figures 1 and 2 trace cumulative incidence C, incidence, and daily growth rate of cumulative incidence $\dot{C} = [C(n+1) - C(n)]/C(n)$, for time step n, for the top eight affected countries (as of 21 March 2020): China, Iran, Italy, South Korea (Supplementary Fig. 1), Spain, Germany, France, USA (Supplementary Fig. 2). The time series begin from the day when the total number of confirmed cases exceeds five. Supplementary Figure 3 traces these time series for Australia. We reiterate that the fraction of imported cases in the overall transmission has been fairly high in Australia, dominating the community transmission, and so we paid particular attention to the daily growth rate in countries where the disease was also introduced predominantly through the air travel (i.e., down-weighting the rates in China and South Korea).

B. Natural history of disease

The natural history of disease is a description of the disease progression over time from exposure to recovery, in a single individual and in the absence of treatment. In the past, the AceMod simulator has been used to model pandemic influenza within Australia, and here we detail modifications of the natural history aimed to account for COVID-19 specifics, captured in AMTraC-19. We define several agent states: susceptible, latent, infectious symptomatic, infectious asymptomatic, and recovered. Consequently, the natural history model considers three distinct phases. The first phase is the latent period during which individuals are infected but unable to infect others, set in the COVID-19 model as two days. The second phase is the period characterised by an exponentially increasing infectivity, from 0% to 100% over three days (see Supplementary Fig. 4). The day on which an individual becomes ill is chosen probabilistically: 30% of agents will change their state to symptomatic one day after exposure, 50% after two days, and the remaining

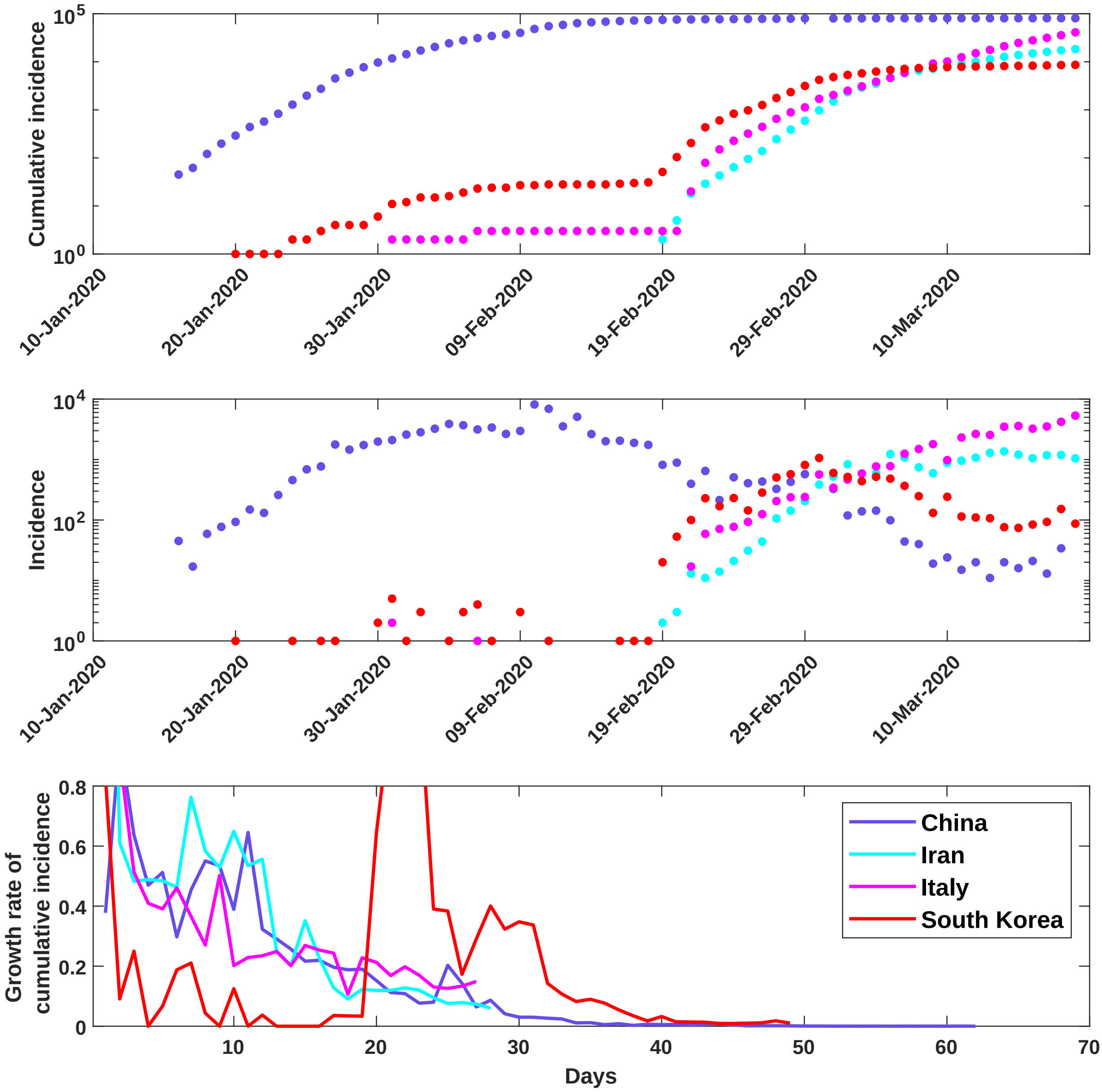

Supplementary Figure 1: **Early epidemic curves: China, Iran, Italy, South Korea.** Cumulative incidence (log scale), incidence (log scale), and daily growth rate of cumulative incidence (up to 19 March 2020). Days: since the day when the total number of confirmed cases exceeded five. Data sources: [3].

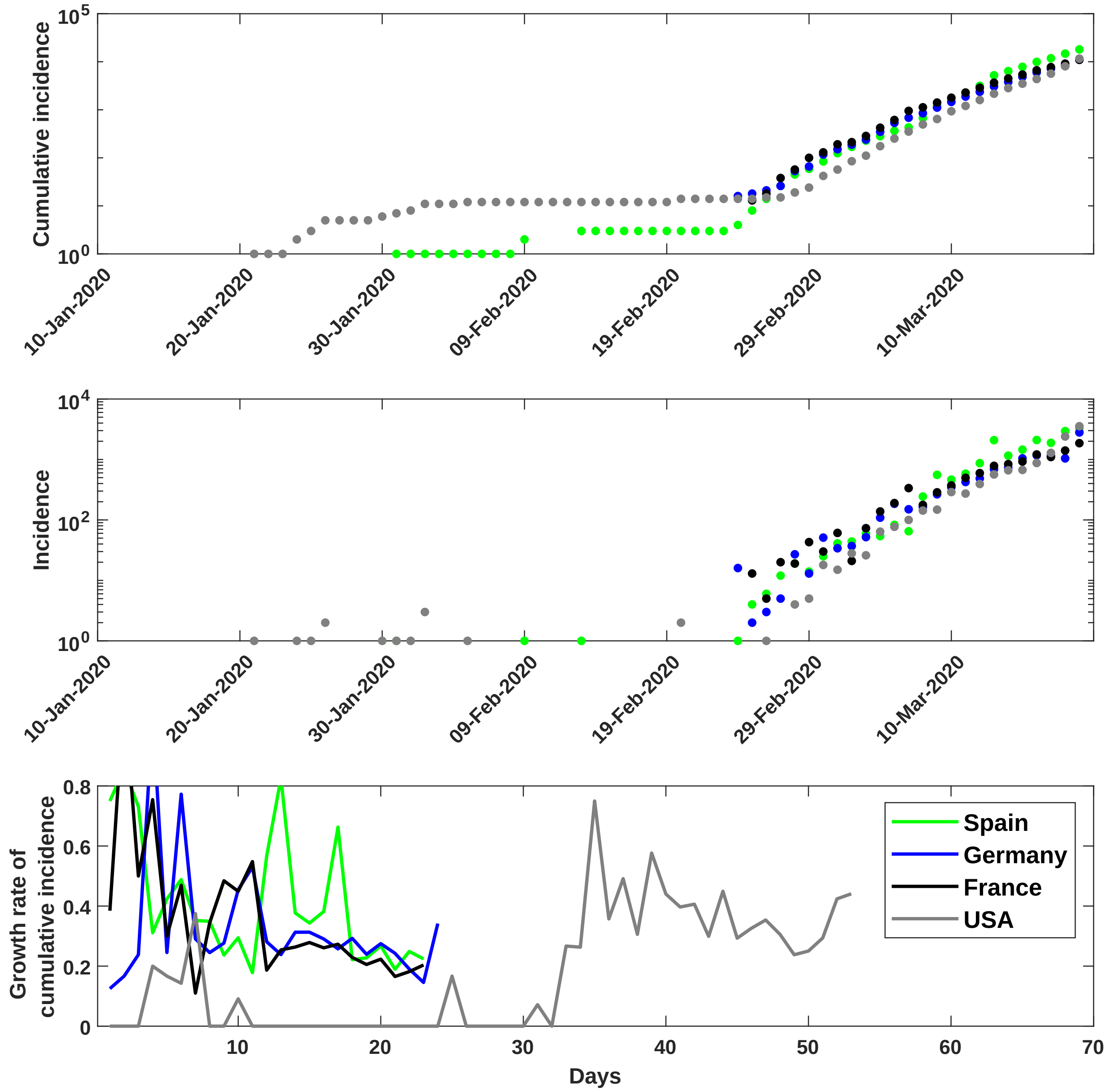

Supplementary Figure 2: **Early epidemic curves: Spain, Germany, France, USA.** Cumulative incidence (log scale), incidence (log scale), and daily growth rate of cumulative incidence (up to 19 March 2020). Days: since the day when the total number of confirmed cases exceeded five. Data sources: [3].

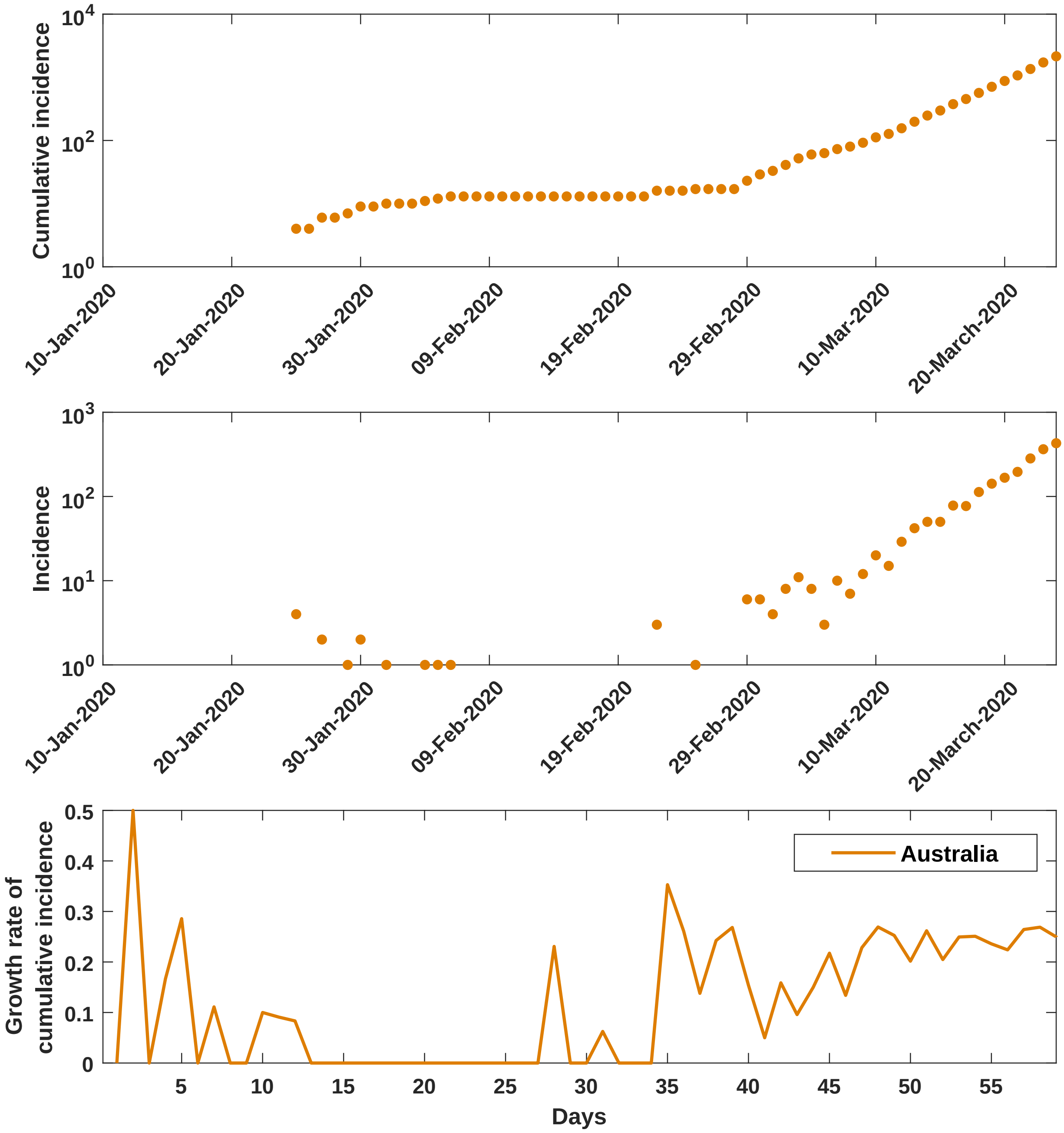

Supplementary Figure 3: **Early epidemic curves: Australia.** Cumulative incidence (log scale), incidence (log scale), and daily growth rate of cumulative incidence (up to 24 March 2020). Days: since the day when the total number of confirmed cases exceeded five. Data sources: [3].

20% will become symptomatic on day three. In order to reflect the presence of mildly symptomatic cases, the model does not follow a canonical definition of an incubation period (the period between exposure to an infection and the appearance of the first symptoms). Instead, it distributes the onset of symptoms across the agents, and increases their infectivity to a peak over a number of days. The time to peak, five days, is chosen to align with an empirically estimated average incubation period, while mild symptoms may be detectable even before the peak. Upon reaching its peak, the infectivity decreases linearly over 12 more days (third phase), until the recovery, with immunity, occurs after 17 days. Finally, we assume that asymptomatic cases are 30% as infectious as symptomatic cases. Unlike influenza, where we assume that the asymptomatic fraction is the same for adults as for children, for the SARS-COV-2 coronavirus we assume that while 67% of adult cases are symptomatic, a significantly lower fraction (13.4%) is symptomatic in children.

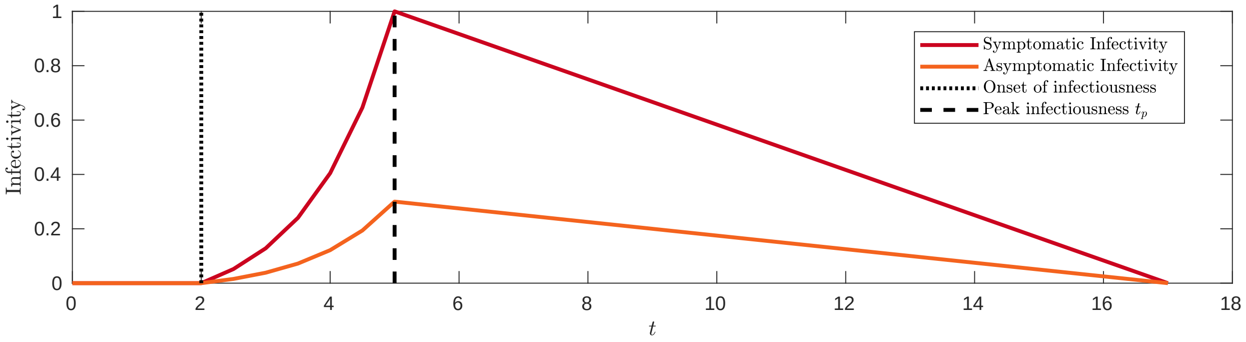

Supplementary Figure 4: **Model of the natural history of COVID-19.** Profile of the infectivity, for both symptomatic and asymptomatic cases. After two days, individuals become infectious, with the infectivity rising exponentially until its peak at five days. After this peak, the infectivity linearly decreases, with full recovery occurring at 17 days. At comparable points within the natural history of disease, asymptomatic individuals are 30% as infectious as symptomatic individuals.

C. Transmission model and reproductive number

The primary dynamics of AMTraC-19 are the infection transmissions. At each time-step the simulator determines the probability of infection for an individual, based on the infection levels in each of their mixing contexts. At each time step we consider all daytime or all nighttime contexts. Let $X_i(n)$ be a random variable describing the state of individual i at time step n. At each time step we calculate $p_i(n) = P(X_i(n) = \text{LATENT}|X_i(n-1) = \text{SUSCEPTIBLE})$, the probability that a susceptible individual is infected at n. Each individual belongs to a number of mixing groups with which an agent interacts, denoted $g \in \mathcal{G}_i(n)$, as well as an associated static set of agents $\mathcal{A}_g$. We define the context-dependent probability $p^g_{j\to i}$ that infectious individual j infects susceptible individual i in context g in a single time step. The probability that a susceptible agent i is infected at a given time step n is thus calculated as:

$$p_i(n) = 1 - \prod_{g\in G_i(n)} \left[\prod_{j\in\mathcal{A}_g\setminus i} (1 - p^g_{j\to i}(n)) \right]. \tag{1}$$

We also define a scaling factor κ (proportional to the reproductive number R_0), as a free parameter which allows us to vary the contagiousness of simulated epidemic scenarios:

$$p^g_{j\to i}(n) = \kappa \, f(n - n_j \mid j) \, q^g_{j\to i} \tag{2}$$

where n_j denotes the time when agent j becomes infected, and $q^g_{j\to i}$ is the probability of transmission from agent j to i at the infectivity peak, derived from the transmission or contact rates. The function $f : \mathbb{N} \to [0,1]$ represents the infectivity of case j over time: $f(n - n_j \mid j) = 0$ when $n < n_j$, implementing the profile shown in Supplementary Fig. 4.

Supplementary Table 1: The input parameters x_i and output variables y_j: local sensitivity analysis with the response $|F_{i,j}|$. Source data are provided as Supplementary Data 1.

Parameter	Default	Range	R_0 (y_1)		T_{gen} (y_2)		$\dot{C}$ (y_3)		A_c (y_4)	
			$\mu^*_{i,1}$	$\sigma_{i,1}$	$\mu^*_{i,2}$	$\sigma_{i,2}$	$\mu^*_{i,3}$	$\sigma_{i,3}$	$\mu^*_{i,4}$	$\sigma_{i,4}$
Time-to-peak, days (x_1)	5	$[4, 7]$	0.47	0.61	1.32	1.27	0.09	0.01	0.002	0.0002
Recovery period, days (x_2)	12	$[7, 21]$	2.79	1.13	5.50	0.66	0.08	0.01	0.014	0.0005
Asymptomatic infectivity (x_3)	0.3	$[0.05, 0.45]$	0.69	0.75	2.42	1.48	0.10	0.01	0.004	0.0004
Symptomatic adults (x_4)	0.669	$[0.5, 0.8]$	0.83	0.60	0.65	0.54	0.06	0.01	0.033	0.0001
Symptomatic children (x_5)	0.134	$[0.05, 0.25]$	0.43	0.55	0.38	0.45	0.07	0.01	0.085	0.0001

Supplementary Table 2: The input parameters x_i and output variables y_j: global sensitivity analysis with the effect $|F_{i,j}|$. Source data are provided as Supplementary Data 2.

Parameter	Range	R_0 (y_1)		T_{gen} (y_2)		$\dot{C}$ (y_3)		A_c (y_4)	
		$\mu^*_{i,1}$	$\sigma_{i,1}$	$\mu^*_{i,2}$	$\sigma_{i,2}$	$\mu^*_{i,3}$	$\sigma_{i,3}$	$\mu^*_{i,4}$	$\sigma_{i,4}$
Time-to-peak, days (x_1)	$[4, 7]$	0.22	0.14	0.98	0.26	0.04	0.01	0.003	0.0038
Recovery period, days (x_2)	$[7, 21]$	2.73	0.30	5.12	0.48	0.05	0.02	0.015	0.0165
Asymptomatic infectivity (x_3)	$[0.05, 0.45]$	0.88	0.41	2.19	0.81	0.08	0.02	0.005	0.005
Symptomatic adults (x_4)	$[0.5, 0.8]$	0.94	0.36	0.89	0.55	0.02	0.01	0.042	0.027
Symptomatic children (x_5)	$[0.05, 0.25]$	0.10	0.11	0.12	0.09	0.02	0.01	0.089	0.0117

This model assumes that for all contexts, the probabilities of infection over a given time period are known. In cases where this information is unavailable, we instead utilise contact rates reported and calibrated in previous studies. Thus, a majority of the transmission and contact probabilities follow previous work on pandemic influenza [4, 5, 6, 7, 8, 9], see Supplementary Tables 7 and 8 in Appendix E. Full details regarding their application can be found in [8].

In this study we used "the attack rate pattern weighted index case" method to calculate R_0 [10, 11]. The method is based on age-specific attack rates, computed as averages over many simulation instances, in order to reduce the bias in determining a typical index case, present due to population heterogeneity. As argued in [12, 11], given the correlation between age group and population structure, the age-stratified weights, assigned to secondary cases produced by a sample of index cases, improve the estimation of the reproductive number R_0. Possible outliers were removed by the interquartile (IQR) method, using 1.5 IQR rule, which makes no assumptions about an underlying distribution. Five age groups were used: [0–4, 5–18, 19–29, 30–64, 65+], with the age-dependent attack rates [0.02, 0.04, 0.18, 0.58, 0.18], producing the corresponding age-dependent reproductive numbers: [1.16, 3.44, 2.63, 2.65, 3.35], with the weighted average of the adjusted reproductive number $R_0 = 2.77$, with 95% CI [2.73, 2.83], constructed from the bias corrected bootstrap distribution (sample size 6,315).

D. Results of sensitivity analysis

D.1. Sensitivity of the model

Results of the local sensitivity analysis are summarised in Supplementary Table 1. The analysis shows that the mean values $\mu^*_{2,1}$ and $\mu^*_{2,2}$, measuring the influence of the recovery period on R_0 and T_{gen} respectively, are larger than the means of the other responses $|F_{i,1}|$ and $|F_{i,2}|$. This indicates that the reproductive ratio and the generation period are most sensitive to changes in the recovery period (x_2). Given the range of the recovery period, varied between 7 and 21 days, each discretisation step $\Delta = 0.1$ corresponds to the

recovery period's change of 1.4 days. For each 1.4-day variation, the reproductive ratio changes by 0.279 on average, resulting in the mean response value $\mu^*_{2,1} = 2.79$. Over the ten steps, these variations extend the reproductive ratio by approximately 0.2 to 0.4 per step, from $R_0 = 1.81$ ($x_2 = 7$ days) to $R_0 = 4.59$ ($x_2 = 21$ days), mostly linearly, as shown in Supplementary Fig. 5.b. Similarly, these variations (linearly) extend the generation period by approximately 0.4 to 0.6 per step, from $T_{gen} = 5.51$ ($x_2 = 7$ days) to $T_{gen} = 11.01$ ($x_2 = 21$ days), Supplementary Fig. 5.b. For each discretisation step, the estimates of R_0 and T_{gen} are produced by "the attack rate pattern weighted index case" method described in section C, with $n = 6,655$ runs on average.

Changes in the other input parameters result in smaller effects on R_0 and T_{gen}, as shown in Supplementary Fig. 5. Overall, despite the sensitivity of the first two output variables to changes in the recovery period (x_2), their variations are within the expected ranges, demonstrating robustness of the model in terms of the reproductive ratio and the generation period.

The other two output variables, the daily growth rate of cumulative incidence at day 50, $\dot{C}$, and the attack rate in children, A_c, show small sensitivity to all input parameters, indicated by the low means $\mu^*_{i,3}$ and $\mu^*_{i,4}$. The asymptomatic infectivity (x_3) is the parameter influencing the growth rate $\dot{C}$ slightly more than other inputs. In response to varying x_3, the daily growth rate at day 50 changes between 0.11 and 0.19, which is an acceptable range, see Supplementary Fig. 5.c. Not surprisingly, the fraction of symptomatic cases among children (x_5) is the parameter with the highest effect on the children attack rate A_c. When x_5 is varied, the attack rate in children changes between 2% and 11%, again within an acceptable range, see Supplementary Fig. 5.e. Thus, all input parameters are weakly influential with respect to output variables y_3 and y_4.

Supplementary Figure 5 shows results of the sensitivity analysis of the model, in terms of five input parameters: the time-to-peak (days, x_1), the recovery period (days, x_2), the probability of transmission for asymptomatic agents. i.e., asymptomatic infectivity (x_3), the fraction of symptomatic cases in adults, i.e., symptomatic adults (x_4), and the fraction of symptomatic cases in children, i.e., symptomatic children (x_5). For each of the input parameters, we trace two output variables which are most affected by this specific input, selected based on ranking of responses $|F_{i,j}|$ using the means $\mu^*_{i,j}$, as reported in Supplementary Table 1. These dependencies are mostly linear, and the output variables are bounded within their anticipated ranges, indicating robustness of the model.

Supplementary Table 2 summarises results of the global sensitivity analysis using the Morris method, carried out with $r = 20$ repeats and $k = 5$ inputs, resulting in 120 parameter combinations, i.e., $r(k + 1)$, with inputs varied over $l = 10$ discretisation levels. When estimating R_0 and T_{gen} for each parameter combination, we use "the attack rate pattern weighted index case" method described in section C, with $n = 6,702$ runs on average. For other two output variables $\dot{C}$ and A_c, we run simulations $m = 10$ times for each parameter combination, averaging the results over these runs before computing the sensitivity effects.

In concordance with the LSA, the reproductive ratio R_0 and the generation period T_{gen} are most sensitive to changes in the recovery period, but also stay within the expected ranges (e.g., R_0 varies between 1.33 and 4.96, and T_{gen} varies between 3.78 and 11.79). The daily growth rate of cumulative incidence at day 50, $\dot{C}$, and the attack rate in children, A_c, show small global sensitivity to all input parameters, despite strong parameter interactions affecting A_c, as evidenced by the higher global $\sigma_{i,4}$.

In summary, the analysis shows that the model is robust to changes in the input parameters, with the highest sensitivity detected in the reproductive ratio and the generation period, in response to the recovery period. Even for the most affected variables, the resulting variations are limited within their expected ranges.

D.2. Sensitivity of the model outcomes

We also investigate whether the model outcomes are sensitive with respect to three context-dependent micro-distancing levels: within households, community, and workplace/school environments. Two specific targets are considered:

(i) the epidemic dynamics traced along 90% SD compliance,
(ii) the transition across the levels of SD compliance, in the range between 70% and 80% levels.

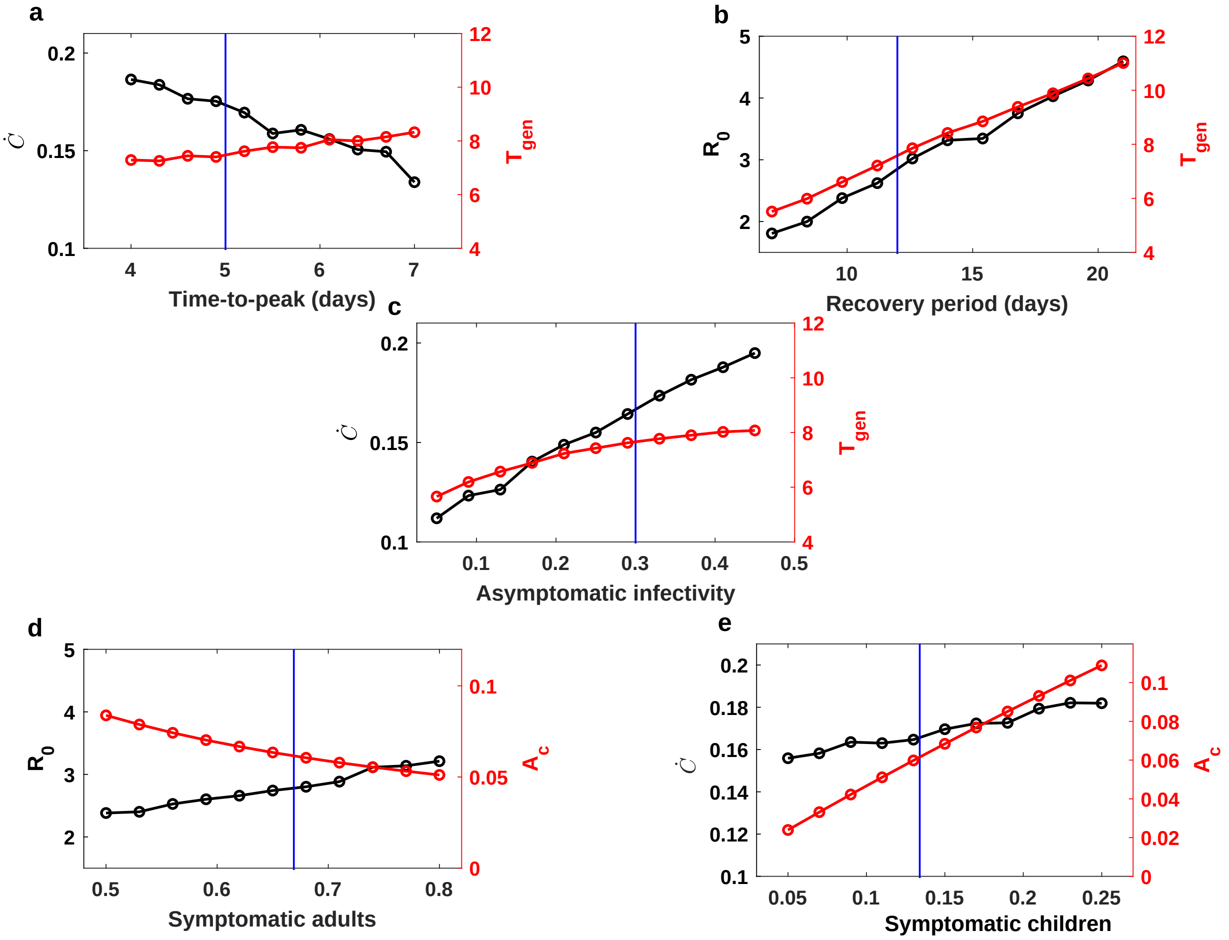

Supplementary Figure 5: **Local sensitivity dependencies of the two most affected output variables for each input parameter.** For each x_i, the output variables y_j with the two highest mean values $\mu^*_{i,j}$ are shown, cf. Supplementary Table 1. The default value of each input parameter is shown with a vertical line. Source data are provided as Supplementary Data 1.

The epidemic dynamics traced along 90% SD compliance is of primary relevance (cf. Fig. 3 of the main manuscript), and so sensitivity of the corresponding outcomes, registered at the end of suppression, is important to establishing the applicability range of the model. The transition in the range between 70% and 80% levels is our main policy-informing result (cf. Fig. 4 of the main manuscript), and its robustness is crucial for our study. For each target, the output variables of interest include the prevalence and cumulative incidence, registered at the end of simulated suppression period.

Each level of micro-distancing is varied by 5% within a 50% range, in proximity of the default values, specified in Supplementary Tables 3 and 4. Using discretisation step $\Delta = 0.1$ and 10 runs per step, we compute the corresponding responses of prevalence and cumulative incidence under 90% SD compliance (Supplementary Table 3), as well as the responses of the difference $\ominus$ between the outcomes under 70% and 80% SD compliance, for both prevalence and cumulative incidence (Supplementary Table 4).

We conclude that both targets are much more sensitive to variations in the micro-distancing levels within the workplace/school environments, and least sensitive to micro-distancing within households. Importantly, the sensitivity dependencies are linear around the default values of input parameters, as shown in Supplementary Figures 6 and 7. This indicates that the model outcomes quantifying the contribution of social macro-distancing to the disease control are robust to the levels of micro-distancing, within certain levels. The onset of non-linearity, seen in Supplementary Figures 6.b and Fig. 6.c, as well as in Supplementary Fig-

Supplementary Table 3: Local sensitivity of the epidemic dynamics under 90% SD compliance to micro-distancing levels. Source data are provided as Supplementary Data 1.

Level of	Default	Range	Prevalence		Cumulative Incidence	
micro-distancing in			μ^*, $\times 10^3$	σ, $\times 10^3$	μ^*, $\times 10^3$	σ, $\times 10^3$
households	100%	[75%, 125%]	0.400	0.061	7.919	1.009
community	50%	[25%, 75%]	2.449	0.291	15.966	1.895
workplace/schools	0%	[0%, 50%]	3.168	2.157	84.106	5.017

Supplementary Table 4: Local sensitivity of the transition between 70% and 80% SD levels to micro-distancing levels. Source data are provided as Supplementary Data 1.

Level of	Default	Range	$\ominus$ Prevalence		$\ominus$ Cumulative Incidence	
micro-distancing in			μ^*, $\times 10^3$	σ, $\times 10^3$	μ^*, $\times 10^3$	σ, $\times 10^3$
households	100%	[75%, 125%]	11.815	1.075	38.739	3.901
community	50%	[25%, 75%]	16.089	2.511	46.822	5.539
workplace/schools	0%	[0%, 50%]	175.140	26.454	327.180	51.540

Supplementary Table 5: Global sensitivity of the epidemic dynamics under 90% SD compliance to micro-distancing levels. Source data are provided as Supplementary Data 2.

Level of	Range	Prevalence		Cumulative Incidence	
micro-distancing in		μ^*, $\times 10^3$	σ, $\times 10^3$	μ^*, $\times 10^3$	σ, $\times 10^3$
households	[75%, 125%]	0.463	0.670	4.550	3.020
community	[25%, 60%]	0.951	0.658	8.026	3.397
workplace/schools	[0%, 25%]	0.900	1.067	5.980	3.937

Supplementary Table 6: Global sensitivity of the transition between 70% and 80% SD levels to micro-distancing levels. Source data are provided as Supplementary Data 2.

Level of	Range	$\ominus$ Prevalence		$\ominus$ Cumulative Incidence	
micro-distancing in		μ^*, $\times 10^3$	σ, $\times 10^3$	μ^*, $\times 10^3$	σ, $\times 10^3$
households	[75%, 125%]	6.162	4.740	14.732	12.136
community	[25%, 60%]	10.982	4.530	20.825	8.769
workplace/schools	[0%, 25%]	17.749	8.248	38.416	16.504

ures 7.b and 7.c, marks the range of applicability in terms of the corresponding micro-distancing parameters. Specifically, the levels of micro-distancing within the workplace/school environments should not exceed 25% (Supplementary Figures 6.c and 7.c), and within the community should stay below 60% (Supplementary Figures 6.b and 7.b), as going beyond these levels increases the sensitivity of the results in a non-linear fashion.

This is further confirmed by the global sensitivity analysis carried out using the Morris method, applied to reduced parameter ranges for the community and workplace/school environments. For each target, the analysis uses $r = 20$ repeats and $k = 3$ inputs (varied over $l = 10$ discretisation levels), resulting in $80 = r(k + 1)$ parameter combinations, each simulated $m = 10$ times. These results, summarised in Supplementary Tables 5 and 6, show that the targets are least sensitive to micro-distancing within households. Another notable observation is that there are limited interactions among micro-distancing parameters, as evidenced by moderate values of σ. Importantly, the model outcomes are robust to globally varying micro-distancing in all social contexts, when these variations are within the identified ranges of applicability.

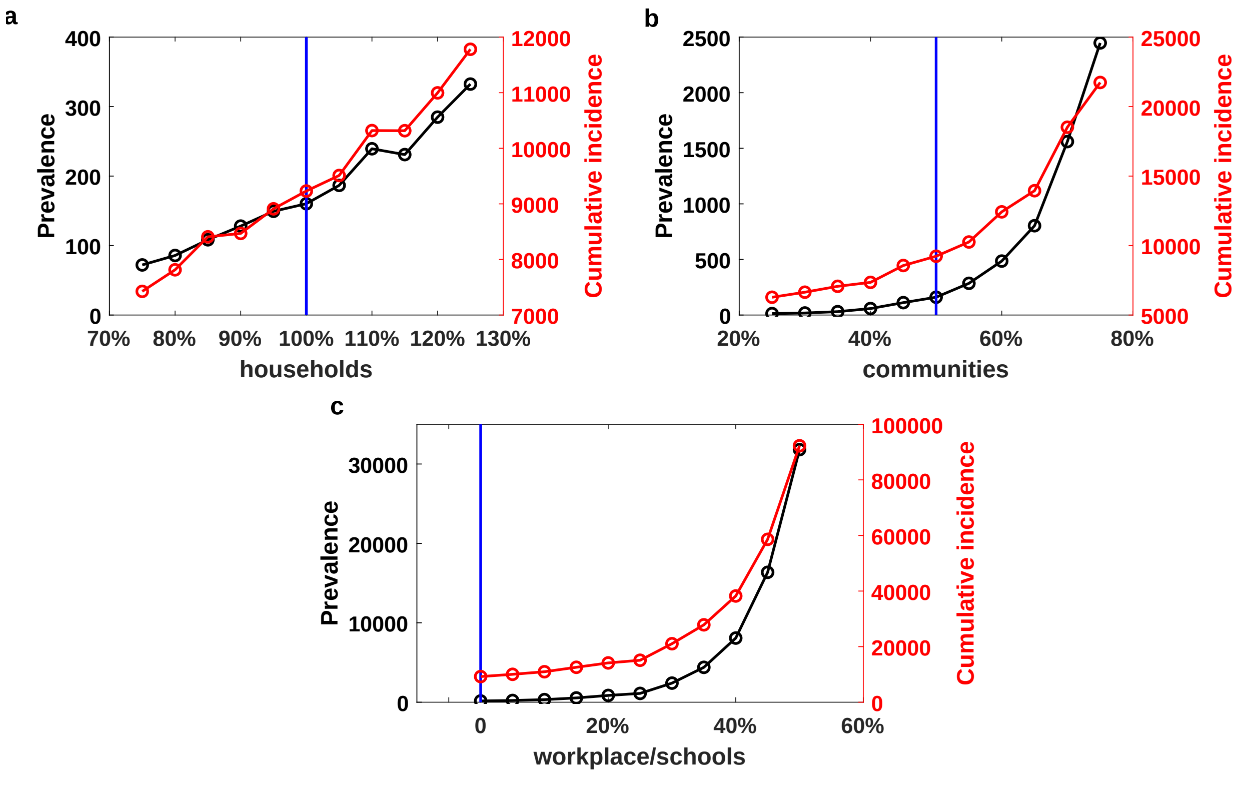

Supplementary Figure 6: **Local sensitivity of prevalence and cumulative incidence.** The sensitivity is traced for 90% SD compliance, coupled with case isolation, home quarantine, and international travel restrictions, to changes in micro-distancing levels in **a** households, **b** community, **c** workplace/school environments. The default value of each input parameter is shown with a vertical line. Source data are provided as Supplementary Data 1.

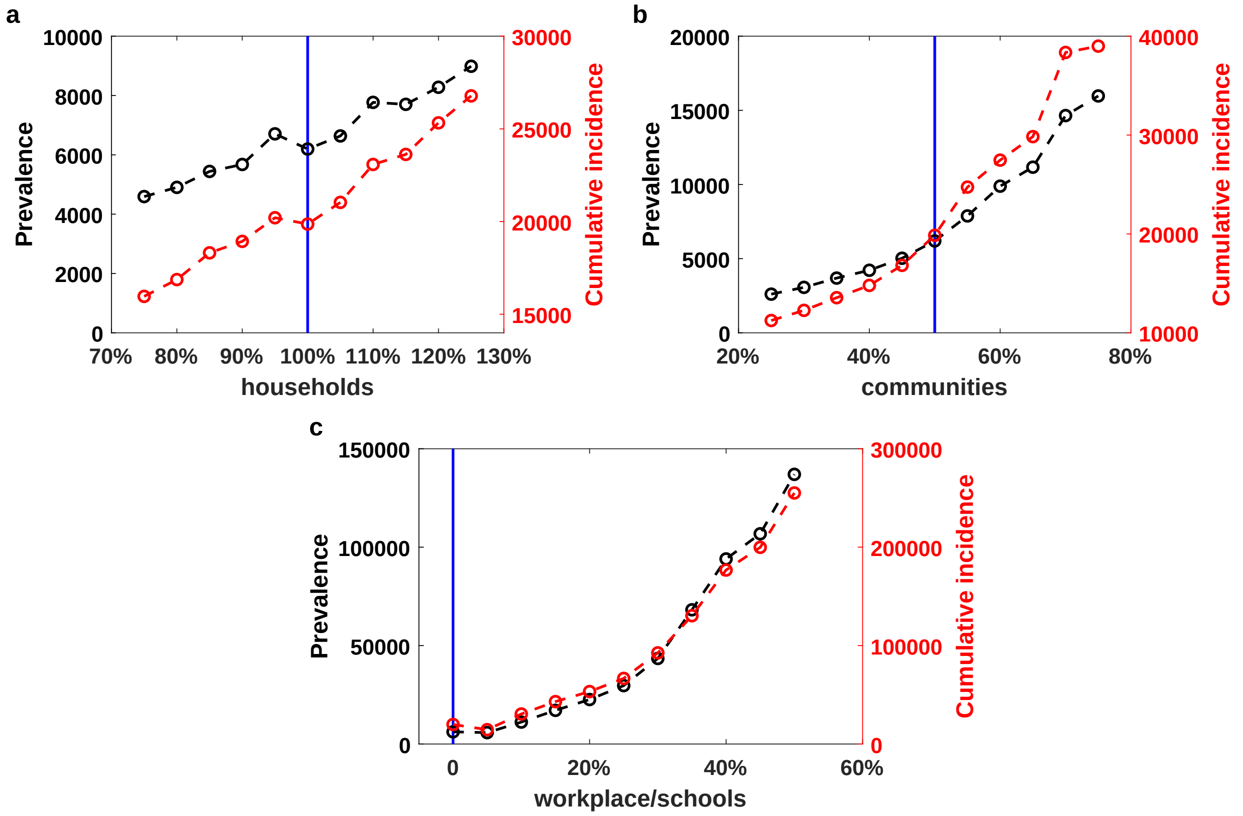

Supplementary Figure 7: **Local sensitivity of changes ⊖ in prevalence and cumulative incidence**. The sensitivity is traced across 70% and 80% SD levels, coupled with case isolation, home quarantine, and international travel restrictions, to changes in micro-distancing levels in **a** households, **b** community, **c** workplace/school environments. The default value of each input parameter is shown with a vertical line. Source data are provided as Supplementary Data 1.

E. Transmission and contact probabilities

Following [8], with some minor adjustments, the contact and transmission probabilities are given in Supplementary Tables 7 and 8, respectively.

F. Population generation, demographics and mobility

Prior to the AMTraC-19 simulations, a surrogate population is generated to match coarse-grained distributions arising from the 2016 Australian census, published by the Australian Bureau of Statistics (ABS). In generating this surrogate population, we use Statistical Areas (SA1 and SA2) level statistics, comprising age, household composition and workplaces. Individuals in the population are separated into 5 different age groups: preschool aged children (0-4), children (5-18), young adults (19-29), adults (30-65) and older adults (65+). Along with these assigned characteristics, individuals are assigned a number of mixing contexts based on the census data. The model uses a discrete-time simulation, where each simulated day is separated into two distinct portions: 'daytime' and 'nighttime'. In the daytime, workplace and school-based mixing are considered, whereas nighttime mixing considers the household transmissions, as well as other local spread at the neighborhood (SA1) and community (SA2) levels.

The population generation begins with the contexts needed for nighttime mixing, which can be thought of as "home regions". The simulation iterates through each SA1, creating a cumulative density function

Supplementary Table 7: Daily contact probabilities $c^g_{j\to i}$ for different contact groups g, reported by [6], reproduced from [8], except for the rates in household clusters. The age is assigned an integer value.

Mixing group g	Infected individual j	Susceptible individual i	Contact probability $c^g_{j\to i}$
Household cluster	Child (≤ 18)	Child (≤ 18)	0.05
	Child (≤ 18)	Adult (≥ 19)	0.05
	Adult (≥ 19)	Child (≤ 18)	0.05
	Adult (≥ 19)	Adult (≥ 19)	0.05
Working Group	Adult (19-64)	Adult (19-64)	0.05
Neighbourhood	Any	Child (0-4)	0.0000435
	Any	Child (5-18)	0.0001305
	Any	Adult (19-64)	0.000348
	Any	Adult (≥ 65)	0.000696
Community	Any	Child (0-4)	0.0000109
	Any	Child (5-18)	0.0000326
	Any	Adult (19-64)	0.000087
	Any	Adult (≥ 65)	0.000174

Supplementary Table 8: Daily transmission probabilities $q^g_{j\to i}$ for different contact groups g, reported by [7], reproduced from [8]. The age is assigned an integer value.

Contact Group g	Infected Individual j	Susceptible Individual i	Transmission Probability $q^g_{j\to i}$
Household size 2	Any	Child (≤ 18)	0.0933
	Any	Adult (≥ 19)	0.0393
Household size 3	Any	Child (≤ 18)	0.0586
	Any	Adult (≥ 19)	0.0244
Household size 4	Any	Child (≤ 18)	0.0417
	Any	Adult (≥ 19)	0.0173
Household size 5	Any	Child (≤ 18)	0.0321
	Any	Adult (≥ 19)	0.0133
Household size 6	Any	Child (≤ 18)	0.0259
	Any	Adult (≥ 19)	0.0107
School	Child (≤ 18)	Child (≤ 18)	0.000292
Grade	Child (≤ 18)	Child (≤ 18)	0.00158
Class	Child (≤ 18)	Child (≤ 18)	0.035

(CDF) describing the size and type of households, based on two dependent probability distributions defined by the ABS. Given this CDF, the procedure begins to randomly generate households, with the generation of agents occurring during this process. Once a household is generated for an SA1, agents are generated to match the size and type of the household (e.g., a single parent family of size four will generate one adult and three children). In order to generate attributes for this surrogate population, the simulation then reads in CDFs describing the population statistics of the given SA, with each of these agents being assigned some attributes based on these population distributions.

Following the population of the home regions, the simulator assigns work and school regions to individuals within the population. This process is based on the "Travel to work" data published by the ABS, which defines a number of individuals N living in home region i and working in region j. In order to satisfy each of these "worker flows", a number of unassigned working-age individuals (19-64 years old) in region i is selected at random and assigned to work in location j. School allocation, on the other hand, is somewhat more complicated as the detailed data about student home locations are not available from the ABS. Instead, we use the available data from the Australian Curriculum, Assessment and Reporting Authority (ACARA), detailing the locations of schools, along with a proximity based model which biases children allocation towards closer schools. More detail about student allocation can be found in previous studies [9].

G. Effects of school closures

Here we compare effects of school closures, added to the case isolation and home quarantine, for two levels of parents' commitment to stay home: 25% and 50%. That is, the proportion of children supervised at home during school closure by one of their randomly chosen parents varies from 25% to 50% (Supplementary Fig. 8). Focussing on the 25% commitment, we also trace the effects of school closures for two specific age groups: children and individuals over 65 years old (Supplementary Figures 9 and 10 respectively).

At this stage we revisit school closures in context of social distancing. As shown in Supplementary Fig. 11, addition of the SC strategy to SD set at 70% generates a reduction in incidence, albeit not lasting and progressing at a higher level than such reductions observed at 80% and 90% SD levels, coupled with school closures. This suggests that another potential but transient benefit of school closures is that it may "compensate" for about 10% lack of SD compliance.

H. Model validation

H.1. A delayed introduction of strong social distancing measures

On 21 March 2020, the number of confirmed COVID-19 cases in Australia crossed 1,000. This coincided with the ban on all international arrivals of non-residents, non-Australian citizens, put in place the night before. The primary scenario considered in this study introduces a social distancing policy, at varying degrees of compliance, triggered by crossing the threshold of 2,000 confirmed cases, exceeded in Australia three days later, on 24 March 2020, when strong measures (e.g., closures of non-essential services and places of social gathering) have been introduced. The primary scenario traced at 90% SD, coupled with case isolation and home quarantine, is well-aligned with the actual epidemic timeline in Australia, as shown in Fig. 3 of the main manuscript, especially in terms of prevalence (Fig. 3.b) and cumulative incidence (Fig. 3.c). The actual daily incidence data (Fig. 3.a) are more noisy, having been affected, in particular, by separate clusters linked to infected cruise ships passengers. For example, by 18 April 2020, more than 600 COVID-19 cases in Australia, i.e., 10% of total cases at the time, have been linked to the Ruby Princess cruise ship from which 2,700 passengers were allowed to disembark on 19 March [13]. Despite the discrepancy in tracing the daily cases, our model accurately predicted timings of the incidence peak (Fig. 3.a) and prevalence peak (Fig. 3.b). In addition, the actual SD levels vary across time, and have been complemented by other surveillance, distancing and intervention measures, e.g., hotel quarantine of international arrivals, meticulous testing of health care workers, inter-state border closures, etc., which are not part of our model. To re-iterate, the model was calibrated by 24 March 2020, and the comparison across the SD levels pointed to 90% SD as the closest match, but did not change the model parametrization, highlighting its robustness and predictive power.

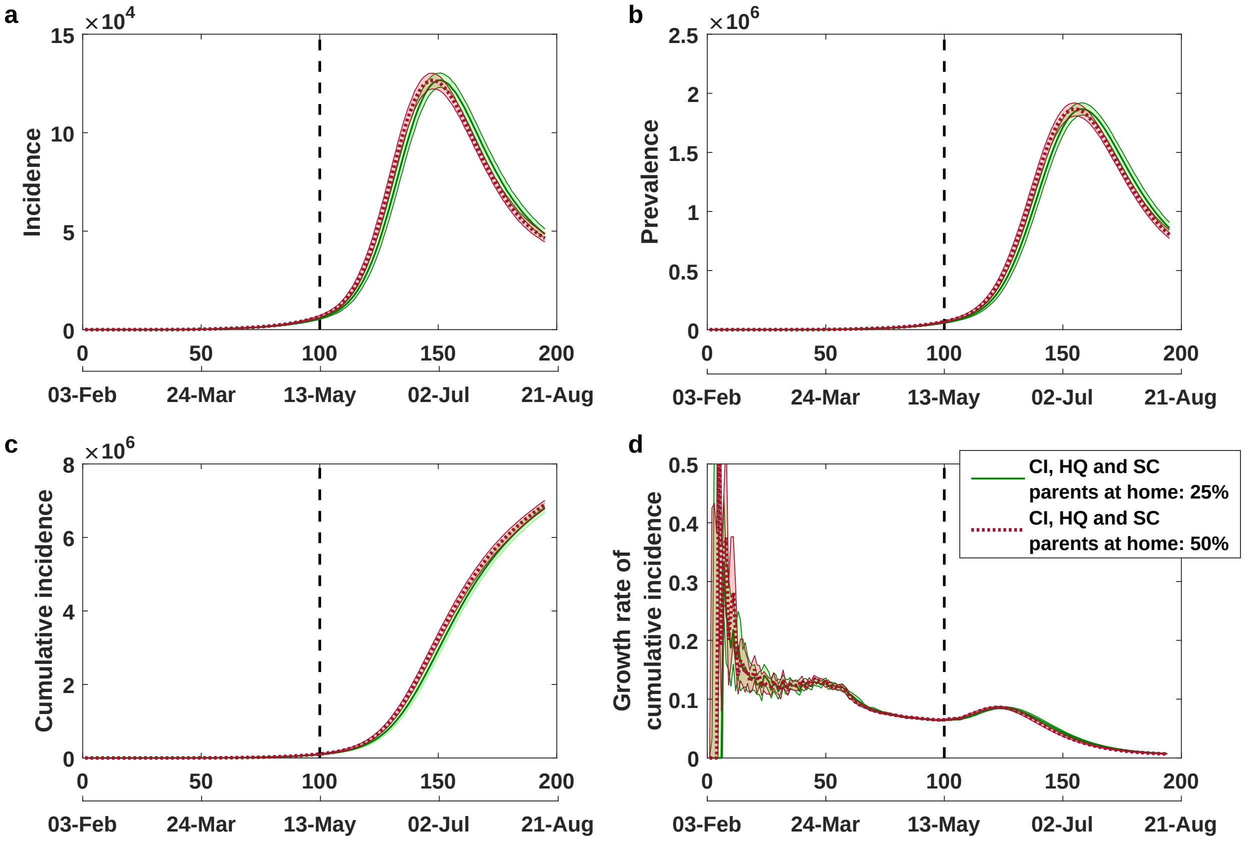

Supplementary Figure 8: **Effects of parents' commitment to stay home during school closures.** Increasing parents' commitment to stay home during school closures (SC) from 25% (solid) to 50% (dashed) does not significantly affect the spread: **a** incidence, **b** prevalence, **c** cumulative incidence, **d** daily growth rate of cumulative incidence, shown as average (solid) and 95% confidence interval (shaded) profiles, over 20 runs. The 95% confidence intervals are constructed from the bias corrected bootstrap distributions. The strategy with school closures (SC) combined with case isolation (CI) and home quarantine (HQ) lasts 49 days (7 weeks), marked by a vertical dashed line. Restrictions on international arrivals are set to last until the end of each scenario. The alignment between simulated days and actual dates may slightly differ across separate runs.

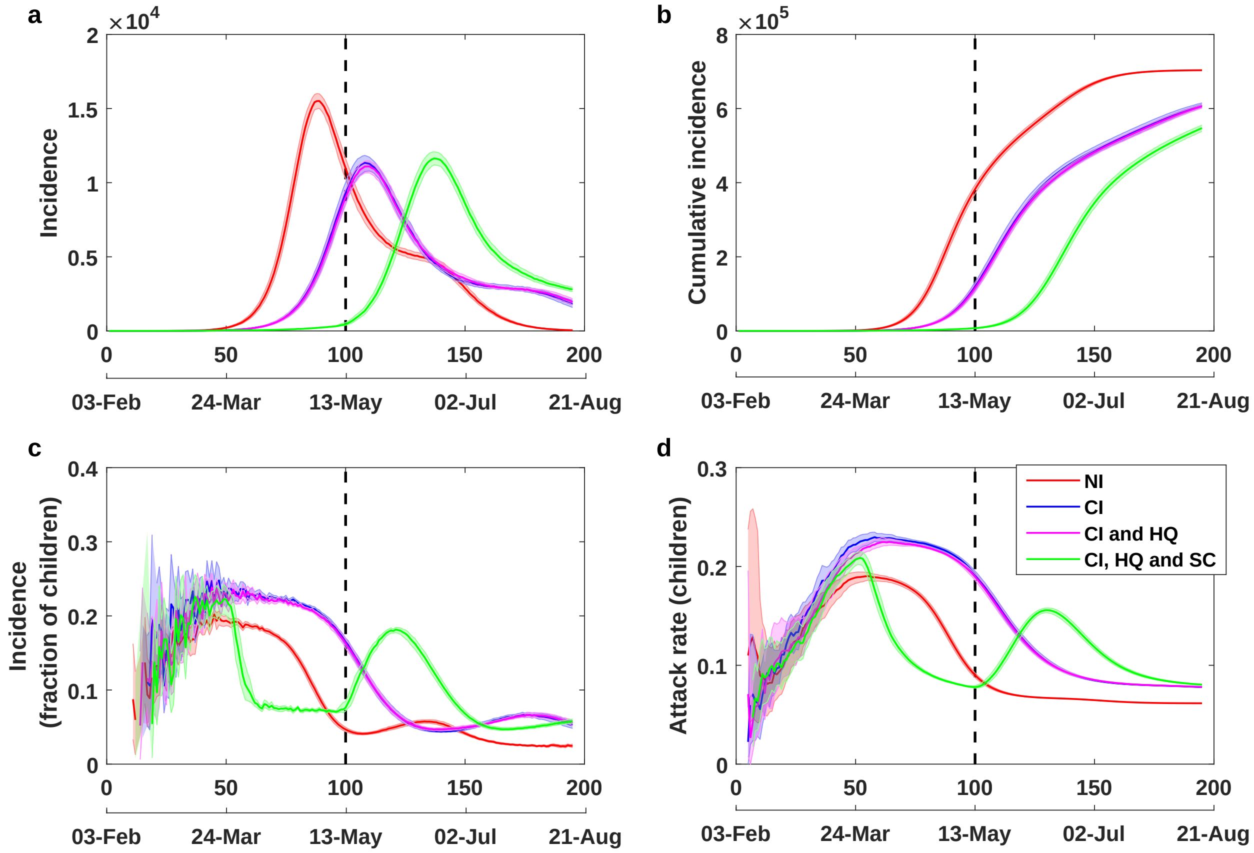

Supplementary Figure 9: **Effects of school closures: children.** School closures (SC) delay incidence peak by four weeks, but increase the fraction of new cases in children around the peak time by 7%, in comparison to case isolation (CI) and home quarantine (HQ), under international travel restrictions. No interventions: NI. Epidemic curves for children: **a** incidence, **b** cumulative incidence, **c** fraction of children in incidence, and **d** fraction of children in cumulative incidence, shown as average (solid) and 95% confidence interval (shaded) profiles, over 20 runs. The 95% confidence intervals are constructed from the bias corrected bootstrap distributions. The strategy with school closures combined with case isolation and home quarantine lasts 49 days (7 weeks), marked by a vertical dashed line. Restrictions on international arrivals are set to last until the end of each scenario. The alignment between simulated days and actual dates may slightly differ across separate runs.

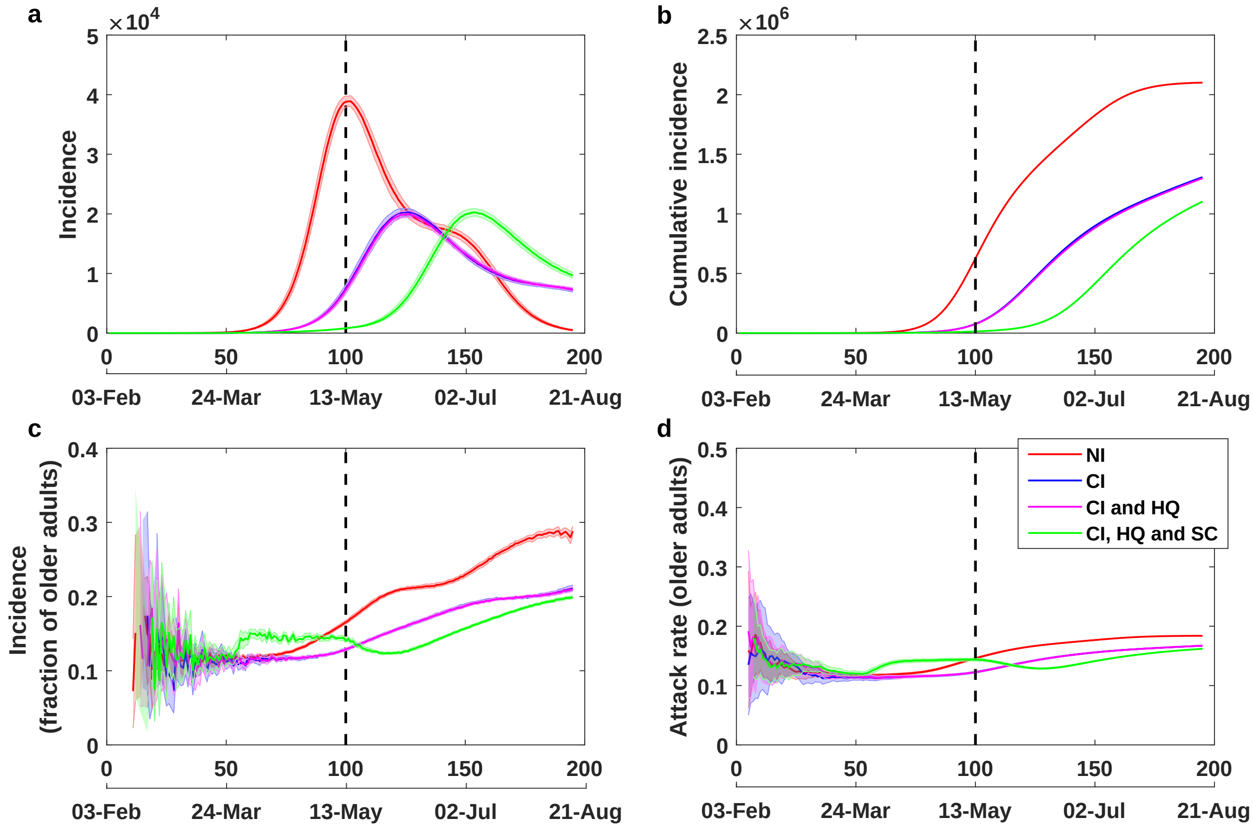

Supplementary Figure 10: **Effects of school closures: older adults.** School closures (SC) delay incidence peak by four weeks, but do not affect new cases for older adults, in comparison to case isolation (CI) and home quarantine (HQ), under international travel restrictions. No interventions: NI. Epidemic curves for older adults: **a** incidence, **b** cumulative incidence, **c** fraction of older adults in incidence, and **d** fraction of older adults in cumulative incidence, shown as average (solid) and 95% confidence interval (shaded) profiles, over 20 runs. The 95% confidence intervals are constructed from the bias corrected bootstrap distributions. The strategy with school closures combined with case isolation and home quarantine lasts 49 days (7 weeks), marked by a vertical dashed line. Restrictions on international arrivals are set to last until the end of each scenario. The alignment between simulated days and actual dates may slightly differ across separate runs.

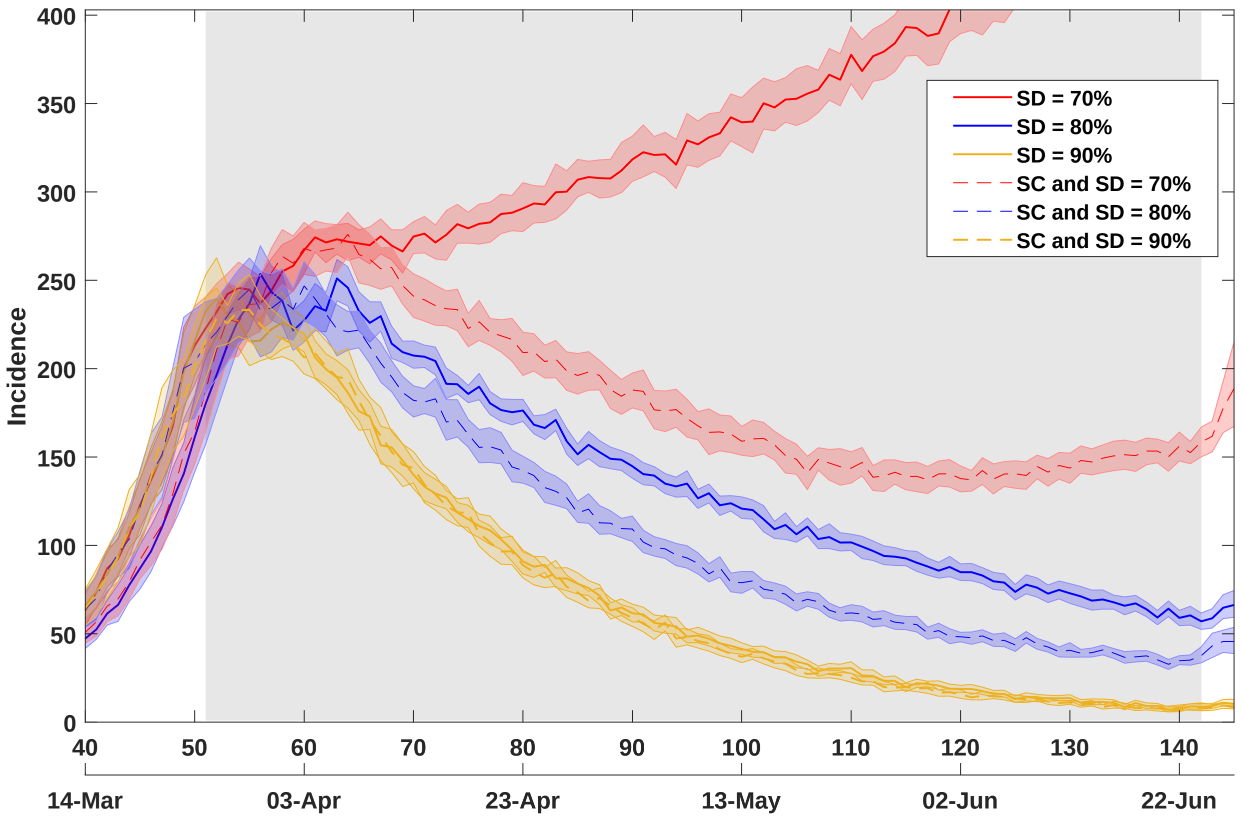

Supplementary Figure 11: **Effects of school closures combined with social distancing.** School closures may temporarily "compensate" for about 10% lack of social distancing (SD) compliance. A comparison of social distancing strategies, coupled with case isolation, home quarantine, international travel restrictions, and school closures (SC) or no school closures, across different compliance levels (70%, 80% and 90%), shown as average (solid) and 95% confidence interval (shaded) profiles, over 20 runs. The 95% confidence intervals are constructed from the bias corrected bootstrap distributions. Duration of each combined SD and SC strategy is set to 91 days (13 weeks), shown as a grey shaded area. Case isolation, home quarantine and restrictions on international arrivals are set to last until the end of each scenario. The alignment between simulated days and actual dates may slightly differ across separate runs.

Supplementary Table 9: The average differences between early and delayed interventions. A comparison between two scenarios: early interventions (threshold 1,000 cases) and delayed interventions (threshold 2,000 cases). Each scenario is evaluated over 20 runs. In each run, a day is recorded when the prevalence decreases below a specified criterion in terms of active cases (ranging from 30 to 50 in increments of 5). Source data are provided as Source Data file.

Criterion	Threshold 1,000		Threshold 2,000		Difference	
(prevalence)	(day)	(std. dev.)	(day)	(std. dev.)	(days)	(std. dev.)
50	148.1	6.708	172.5	7.345	24.4	9.947
45	151.0	8.079	175.4	7.162	24.4	10.796
40	153.7	8.899	177.6	7.229	23.9	11.465
35	156.7	9.183	181.3	7.987	24.6	12.171
30	161.9	9.520	182.4	6.373	20.5	11.456
Average					23.56	11.167

To evaluate a delayed introduction of strong social distancing measures, we compare these two thresholds, separated by three days, while keeping all other parameters unchanged. Referring to Supplementary Fig. 12, a delayed response results in higher epidemic peaks, doubling the prevalence in comparison with the alternative scenario (Fig. 12.b), across different levels of compliance. The cumulative incidence for 90% SD nearly doubles as well, from around 5,000 total cases, to about 9,000 (Supplementary Fig. 12.c).

We also observe that a three-day delay in introducing strong social distancing measures results in an approximately four-week lengthening of the required suppression period, confirmed by separate runs with a longer suppression duration (Supplementary Table 9). The resultant difference (i.e., delay) averages in 23.56 days, with standard deviation of the difference estimated as 11.167 days.

H.2. Forecasting

This model has been used in Australia in a now-casting mode during the period since 24 March 2020. In the simulation timeline, the threshold of 2,000 cases is crossed on day 50, and if this is aligned with 24 March 2020 on the actual timeline, one may see that the incidence along the 90% SD curve starts to reduce from day 59 (aligned with early April 2020), Fig. 3.a of the main manuscript, and the prevalence peak is reached around days 62–65 (aligned with 5–8 April 2020), Fig. 3.b.

The early projections of the timing of actual incidence and prevalence peaks, as well as three-month ahead forecast of the cumulative incidence in Australia to approach the range of 8,000–10,000 total cases, have shown a good accuracy, validating the model. Specifically, the agreement between the actual and simulation timelines appears to be the strongest for 90% SD compliance, applied from 24 March 2020 (i.e., primary scenario with 2,000 cases), following a period of weaker compliance between 21 and 24 of March 2020. The predicted cumulative incidence at the end of the suppression period, which maps to the end of June, averages 9,122 cases with 95% CI [8,898, 9,354], and the range over 20 runs is 8,313 − 10,090 (see Source Data file). The actual number of total cases in Australia on 30 June 2020 is reported as 7,834 [3].

Significant levels of compliance have been confirmed by the Citymapper Mobility Index, which collates the usage of the Citymapper app, a worldwide public transit app and mapping service which integrates data for all urban modes of transport, for planning public transport, walking, cycling, and micromobility data [14]. These data allow for approximating the extent of social distancing compliance, showing, by the 26th March 2020, a reduction of 80% from the normal mobility levels for both Sydney and Melbourne. There was a relatively steep drop in mobility to this level, noting that the number of trips taken by residents of Sydney and Melbourne was around 50% just five days prior. Since this drop to mid-April, the levels of compliance have remained relatively constant at 80–90%, peaking at 90% for both Sydney and Melbourne on the 10th April 2020. Comparable levels of social distancing were also inferred from the anonymised and aggregated mobile phone location data of several million Australians, provided in early April 2020 by Vodafone, a multinational telecommunications company, to the Australian federal government. These data showed a reduction of 83% from the normal mobility levels for Sydney, and 82% for Melbourne [15]. A

national online survey of 1,420 Australian adults, carried out between 18 and 24 March 2020, found that over the last month 93.4% of respondents followed at least one of six avoidance-related behaviors [16]. In addition, the ABS survey taken during 1-6 April 2020, showed that during the preceding four weeks, 88.3% of Australians have been avoiding public spaces (and public events), 98.4% have been keeping distance from people, and 86.6% cancelled personal gatherings (e.g., with friends or family) [17].

We point out that in Australia, the healthcare sector alone comprises about 6% the population, with accommodation and food services reaching up to 3.6%, while transport, postal and warehousing sector occupies 2.6%, and electricity, gas, water and Waste services add another 0.6% [18]. Thus, assuming that a substantial fraction of employees delivering these essential services cannot work from home, the highest level of social distance compliance would not exceed 90%.

I. Comparison of SD compliance levels across several state capitals

Differences between 70% and 90% SD compliance levels are visualised in choropleth maps of four largest Australian Capital Cities: Sydney, Melbourne, Brisbane and Perth (Supplementary Fig. 13). These maps contrast prevalence numbers resulting from these two compliance levels at day 60.

J. Fractions of symptomatic cases across mixing contexts

Supplementary Table 10 summarises fractions of symptomatic cases for the considered scenarios, across mixing contexts: households (HH), household clusters (HC), census districts (CD), statistical areas (SA2), working groups (WG), classrooms (CL), grades (GR), schools (SCH). Notably, a stronger compliance with social distancing, in addition to case isolation and home quarantine, increases the household fraction from 30.48% (the household fraction under case isolation and home quarantine) to 47.79% (the household fraction under full lockdown). This is compensated by the corresponding decreases in the infections acquired at the workplace: from 17.01% to 6.98%, as well as in the school environments: from 12.41% to 6.20%.

Supplementary Table 10: Average context-dependent fractions of symptomatic cases (over 20 runs, rounded to two decimal places), in %. NI: no intervention, CI: case isolation, HQ: home quarantine, SC: school closures, SD: social distancing. For NI, CI, HQ and SC: shown at the end of suppression of SC, i.e., after approximately 102 days (including 49 days of suppression). For SD: shown at the end of suppression of SD, i.e., after approximately 143 days (including 91 days of suppression). The contexts include households (HH), household clusters (HC), census districts (CD), statistical areas (SA2), working groups (WG), classrooms (CL), grades (GR), schools (SCH). Source data are provided as Source Data file.

Scenario	HH	HC	CD	SA2	WG	CL	GR	SCH
NI	17.88	17.21	25.75	13.89	20.46	1.94	1.46	1.40
CI	28.27	15.00	17.14	9.38	17.37	5.12	3.93	3.78
CI+HQ	30.48	14.31	16.65	9.14	17.01	4.95	3.80	3.66
CI+HQ+SC	26.68	17.02	25.57	14.13	16.22	0.14	0.11	0.11
CI+HQ+SD 10%	30.61	14.88	20.87	11.35	16.54	2.32	1.76	1.67
CI+HQ+SD 20%	32.62	14.80	20.13	10.99	15.17	2.53	1.93	1.83
CI+HQ+SD 30%	34.56	14.60	19.28	10.54	13.88	2.86	2.19	2.09
CI+HQ+SD 40%	36.63	14.34	18.36	10.04	12.55	3.20	2.49	2.38
CI+HQ+SD 50%	38.46	14.17	17.90	9.82	11.33	3.29	2.56	2.48
CI+HQ+SD 60%	40.45	14.11	18.17	9.96	9.85	2.92	2.30	2.23
CI+HQ+SD 70%	42.45	14.07	19.01	10.34	8.24	2.32	1.82	1.75
CI+HQ+SD 80%	44.54	13.81	18.97	10.46	7.09	1.99	1.60	1.54
CI+HQ+SD 90%	46.01	13.30	18.33	9.97	6.85	2.17	1.75	1.63
CI+HQ+SD 100%	47.79	12.78	16.99	9.25	6.98	2.45	1.90	1.85

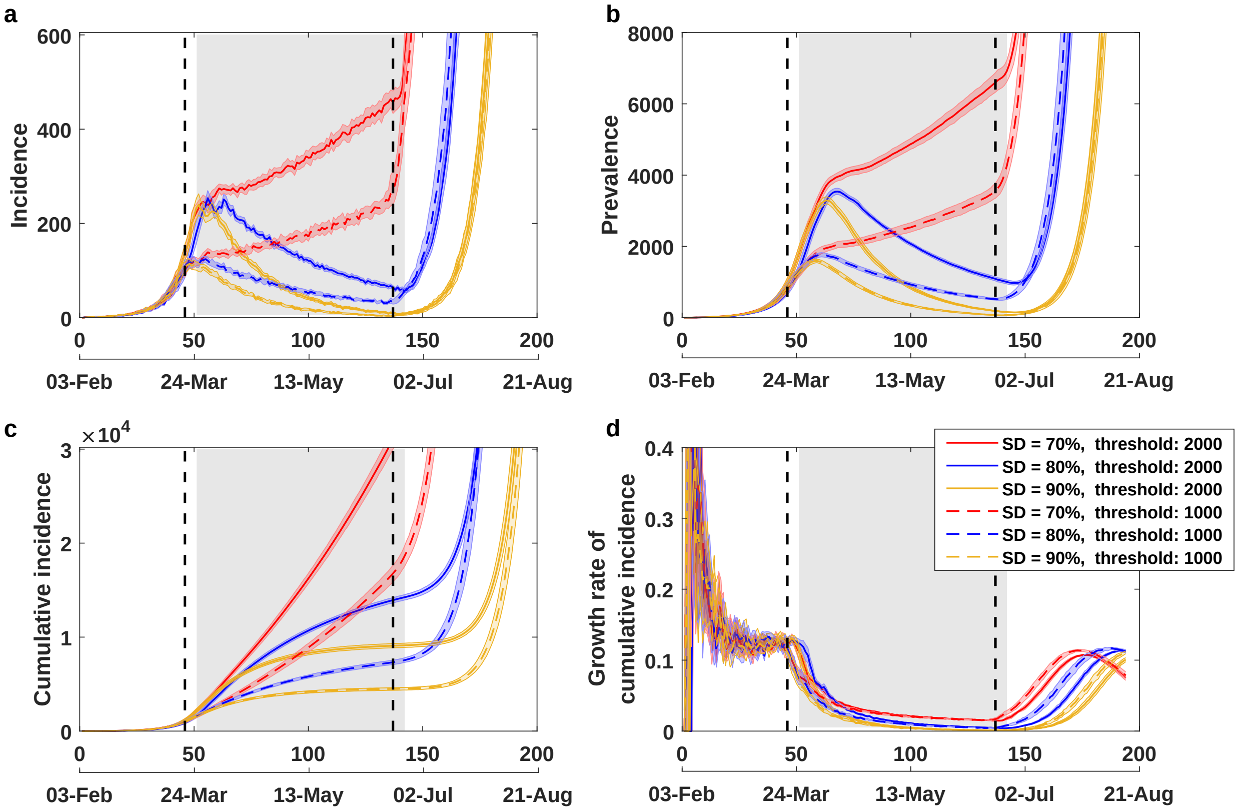

Supplementary Figure 12: **Effects of delays.** A three-day delay in introducing strict social distancing doubles the disease prevalence. A comparison of social distancing (SD) strategies, coupled with case isolation, home quarantine, and international travel restrictions, across different compliance levels (70%, 80% and 90%). Two scenarios are contrasted: primary scenario with the threshold set at 2,000 cases (matching actual numbers on 24 March 2020), and the alternative threshold of 1,000 (matching actual numbers on 21 March 2020). Duration of each SD strategy is set to 91 days (13 weeks), shown as a grey shaded area for the primary threshold (2,000 cases), and with vertical dashed lines for the alternative threshold (1,000 cases). Case isolation, home quarantine, and restrictions on international arrivals are set to last until the end of each scenario. Traces include **a** incidence, **b** prevalence, **c** cumulative incidence, and **d** the daily growth rate of cumulative incidence $\dot{C}$, shown as average (solid) and 95% confidence interval (shaded) profiles, over 20 runs. The 95% confidence intervals are constructed from the bias corrected bootstrap distributions. The alignment between simulated days and actual dates may slightly differ across separate runs.

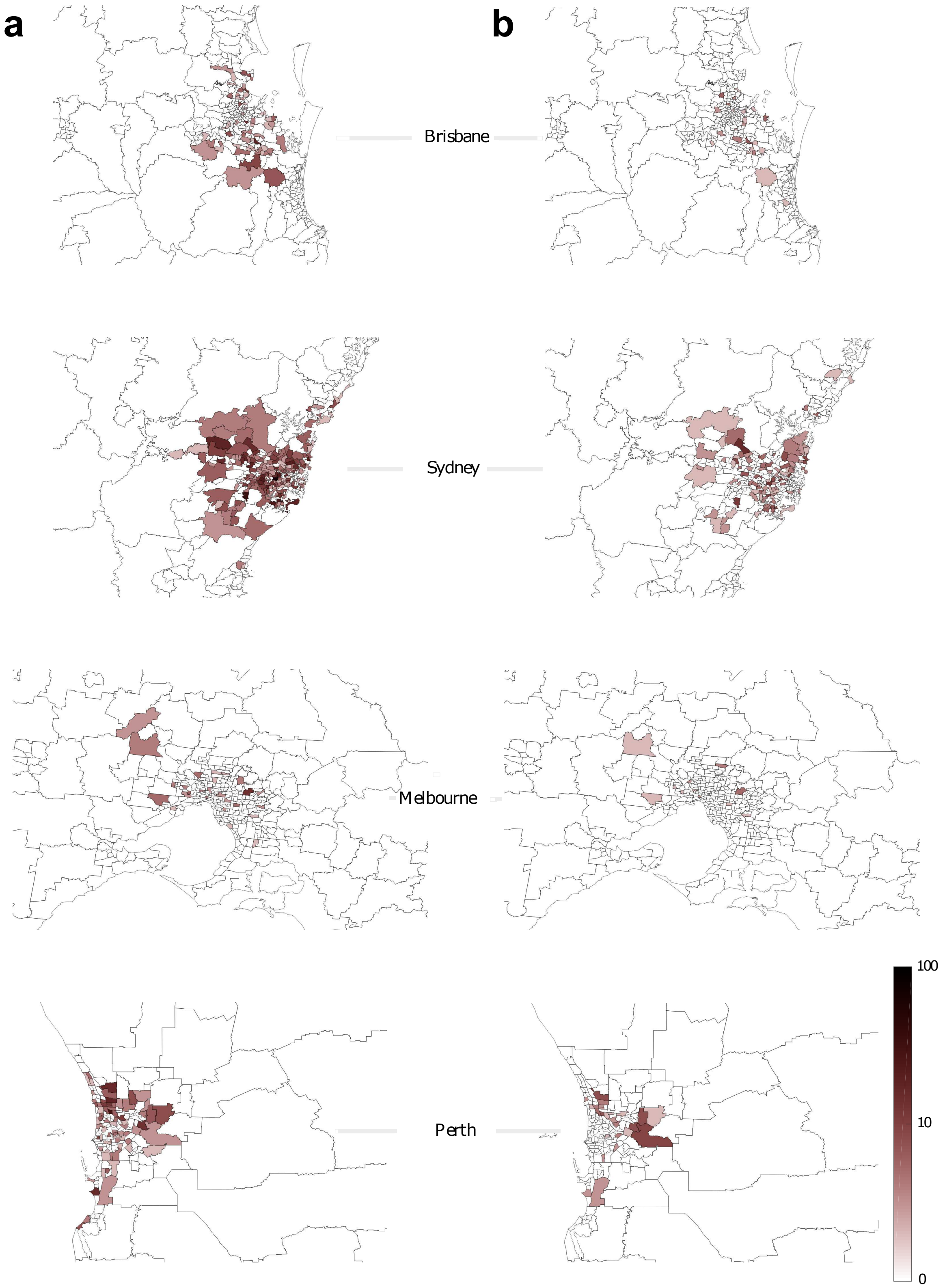

Supplementary Figure 13: **Choropleths of four largest Australian Capital Cities.** Prevalence is shown on a log scale at day 60: **a** 70% SD compliance, and **b** 90% SD compliance.

References

[1] Dong, E., Du, H. & Gardner, L. An interactive web-based dashboard to track COVID-19 in real time. *The Lancet Infectious Diseases* (2020).

[2] Coronavirus COVID-19 global cases: Johns Hopkins University. https://www.arcgis.com/apps/opsdashboard/index.html (2020).

[3] Wikipedia contributors. 2019–20 coronavirus pandemic in mainland China; 2020 coronavirus pandemic in <nation> — Wikipedia, The Free Encyclopedia (2020). [Online; accessed 30-June-2020; nation: Australia, France; Germany; Iran; Italy; South Korea; Spain; the United States].

[4] Halloran, M. E. *et al.* Modeling targeted layered containment of an influenza pandemic in the United States. *Proceedings of the National Academy of Sciences* **105**, 4639–4644 (2008).

[5] Mossong, J. *et al.* Social contacts and mixing patterns relevant to the spread of infectious diseases. *PLoS Medicine* **5**, e74 (2008).

[6] Chao, D. L., Halloran, M. E., Obenchain, V. J. & Longini Jr, I. M. FluTE, a publicly available stochastic influenza epidemic simulation model. *PLoS Computational Biology* **6**, e1000656 (2010).

[7] Cauchemez, S. *et al.* Role of social networks in shaping disease transmission during a community outbreak of 2009 H1N1 pandemic influenza. *Proceedings of the National Academy of Sciences* **108**, 2825–2830 (2011).

[8] Cliff, O. M. *et al.* Investigating spatiotemporal dynamics and synchrony of influenza epidemics in Australia: an agent-based modelling approach. *Simulation Modelling Practice and Theory* **87**, 412–431 (2018).

[9] Zachreson, C. *et al.* Urbanization affects peak timing, prevalence, and bimodality of influenza pandemics in Australia: results of a census-calibrated model. *Science Advances* **4**, eaau5294 (2018).

[10] Germann, T. C., Kadau, K., Longini, I. M. & Macken, C. A. Mitigation strategies for pandemic influenza in the United States. *Proceedings of the National Academy of Sciences* **103**, 5935–5940 (2006).

[11] Zachreson, C., Fair, K. M., Harding, N. & Prokopenko, M. Interfering with influenza: nonlinear coupling of reactive and static mitigation strategies. *Journal of The Royal Society Interface* **17**, 20190728 (2020).

[12] Miller, J. C. Spread of infectious disease through clustered populations. *Journal of the Royal Society Interface* **6**, 1121–1134 (2009).

[13] Klein, A. Australia keeps a lid on COVID-19 – for now. *New Scientist* **246**, 10 (2020).

[14] CityMapper. CityMapper Mobility Index. https://citymapper.com/cmi/. Accessed: 12-04-2020.

[15] Grubb, B. Mobile phone location data used to track Australians' movements during coronavirus crisis, The Sydney Morning Herald. https://www.smh.com.au/technology/mobile-phone-location-data-used-to-track-australians-movements-during-coronavirus-crisis-20200404-p54h09.html. Accessed: 02-04-2020.

[16] Seale, H. *et al.* COVID-19 is rapidly changing: Examining public perceptions and behaviors in response to this evolving pandemic. *PLOS ONE* **15**, e0235112 (2020).

[17] 4940.0 – Household Impacts of COVID-19 Survey, 1-6 Apr 2020. The Australian Bureau of Statistics. https://www.abs.gov.au/AUSSTATS/abs@.nsf/allprimarymainfeatures/4DF23BAE08F75714CA25855B0003B1D9?opendocument (2020). Accessed: 06-06-2020.

[18] Vandenbroek, P. Snapshot of employment by industry, 2019. https://www.aph.gov.au/About_Parliament/Parliamentary_Departments/Parliamentary_Library/FlagPost/2019/April/Employment-by-industry-2019. Accessed: 02-04-2020.
