# Supplementary material for: Modelling transmission and control of the COVID-19 pandemic in Australia
Source: Nat Commun. 2020 Nov 11;11:5710. doi: 10.1038/s41467-020-19393-6 (PMC7659014; doi:10.1038/s41467-020-19393-6)
Supplement: Supplementary file 3 — Descriptions of Additional Supplementary Files [file 41467_2020_19393_MOESM3_ESM.pdf]

Descriptions of Additional Supplementary Files

Supplementary Data 1
Description: Local Sensitivity Analysis of the model and model outcomes (Supplementary Tables 1, 3, 4; Supplementary Figures 5, 6, 7).

Supplementary Data 2
Description: Global Sensitivity Analysis of the model and model outcomes (Supplementary Tables 2, 5, 6).
