# Supplementary material for: Modelling transmission and control of the COVID-19 pandemic in Australia
Source: Nat Commun. 2020 Nov 11;11:5710. doi: 10.1038/s41467-020-19393-6 (PMC7659014; doi:10.1038/s41467-020-19393-6)
Supplement: Supplementary file 6 — Reporting Summary [file 41467_2020_19393_MOESM6_ESM.pdf]

nature research

Corresponding author(s): Mikhail Prokopenko

Last updated by author(s): Oct 2, 2020

Reporting Summary

Nature Research wishes to improve the reproducibility of the work that we publish. This form provides structure for consistency and transparency in reporting. For further information on Nature Research policies, see Authors & Referees and the Editorial Policy Checklist .

Statistics

For all statistical analyses, confirm that the following items are present in the figure legend, table legend, main text, or Methods section.

n/a	Confirmed	
☐	☒	The exact sample size (*n*) for each experimental group/condition, given as a discrete number and unit of measurement
☐	☒	A statement on whether measurements were taken from distinct samples or whether the same sample was measured repeatedly
☒	☐	The statistical test(s) used AND whether they are one- or two-sided<br>*Only common tests should be described solely by name; describe more complex techniques in the Methods section.*
☒	☐	A description of all covariates tested
☐	☒	A description of any assumptions or corrections, such as tests of normality and adjustment for multiple comparisons
☐	☒	A full description of the statistical parameters including central tendency (e.g. means) or other basic estimates (e.g. regression coefficient) AND variation (e.g. standard deviation) or associated estimates of uncertainty (e.g. confidence intervals)
☒	☐	For null hypothesis testing, the test statistic (e.g. *F*, *t*, *r*) with confidence intervals, effect sizes, degrees of freedom and *P* value noted<br>*Give P values as exact values whenever suitable.*
☒	☐	For Bayesian analysis, information on the choice of priors and Markov chain Monte Carlo settings
☒	☐	For hierarchical and complex designs, identification of the appropriate level for tests and full reporting of outcomes
☒	☐	Estimates of effect sizes (e.g. Cohen's *d*, Pearson's *r*), indicating how they were calculated

Our web collection on statistics for biologists contains articles on many of the points above.

Software and code

Policy information about availability of computer code

Data collection	C++ 11 Linux, tested on Ubuntu v16.04 and CentOS release 6.9 (upstream Red Hat 4.4.7-18). Our implementation is based on "Australian Census-based Epidemic Model (ACEMod)" software, invention disclosure with The University of Sydney, CDIP Ref. 2019-123. The COVID-19 modeling component, "Agent-based Model of Transmission and Control of the COVID-19 pandemic in Australia (AMTraC-19)", is a separate invention disclosure, AMTRaC-19, with the University of Sydney, CDIP Ref. 2020-018.<br><br>The compilation script uses GNU Autotools (autoconf 2.69, automake 1.15) and the g++ compiler (GCC) 4.9.3 which are licensed under the GNU General Public License.
Data analysis	MATLAB R2020a

For manuscripts utilizing custom algorithms or software that are central to the research but not yet described in published literature, software must be made available to editors/reviewers. We strongly encourage code deposition in a community repository (e.g. GitHub). See the Nature Research guidelines for submitting code & software for further information.

Data

Policy information about availability of data

All manuscripts must include a data availability statement. This statement should provide the following information, where applicable:

- Accession codes, unique identifiers, or web links for publicly available datasets
- A list of figures that have associated raw data
- A description of any restrictions on data availability

The code and data can be made available to approved bona fide researchers after their host institution has signed a Data Access/Confidentiality Agreement with the University of Sydney. Mediated access will enable code/data to be shared and results to be confirmed without unduly compromising the University's ability to commercialize the software. To the extent that this code/data sharing does not violate the commercialization and licensing agreements entered into by the University of Sydney, the code/data will be made publicly available after the appropriate licensing terms agreed.

Field-specific reporting

Please select the one below that is the best fit for your research. If you are not sure, read the appropriate sections before making your selection.

☒ Life sciences ☐ Behavioural & social sciences ☐ Ecological, evolutionary & environmental sciences

For a reference copy of the document with all sections, see nature.com/documents/nr-reporting-summary-flat.pdf

Life sciences study design

All studies must disclose on these points even when the disclosure is negative.

Sample size

AceMod, the Australian Census-based Epidemic Model, is a simulator comprising over 24 million software agents, each with attributes of an anonymous individual (e.g., age, gender, occupation, susceptibility and immunity to diseases), as well as mobility data, connecting places of residence to schools and workplaces. In addition, the AceMod simulator has integrated layered school attendance data from the Australian Curriculum, Assessment and Reporting Authority (ACARA). The sample size corresponds to the entire population of Australia, as represented in the Australian Bureau of Statistics datasets. In generating this surrogate population, we use Statistical Areas (SA1 and SA2) level statistics, comprising age, household composition and workplaces. Individuals in the simulated population are separated into 5 different age groups: preschool aged children (0-4), children (5-18), young adults (19-29), adults (30-65) and older adults (65+). Along with these assigned characteristics, simulated individuals are assigned a number of mixing contexts based on the 2016 Australian census data. The details are provided in Appendix F.

Data exclusions

Individuals' occupation data were excluded, except for school teachers and students. The simulation of the COVID-19 epidemic transmission within simulated working groups are set independently of the agent occupations (apart from teachers and students), and the presented results do not draw conclusions relevant to the excluded data (e.g., there are no inferences on occupational risks or economic ramifications within sectors).

Replication

AceMod and AMTraC-19 employ a discrete-time and stochastic agent-based model, with multiple simulation runs ensuring replication of the results within indicated bounds. In this study, we used 20 runs for each separate simulated scenario (baseline, case isolation, home quarantine, school closures, social distancing at different levels of compliance from 0.0 to 1.0, in increments of 0.05). For computing reproductive ratio and generation period, we used between 6,274 and 6,413 runs.

For local sensitivity analysis of the model, we used 55 parameter combinations. For local sensitivity analysis of each of the model outcomes, we used 33 parameter combinations. For global sensitivity analysis of the model, we used 120 parameter combinations. For global sensitivity analysis of each of the model outcomes, we used 80 parameter combinations. Each parameter combination was simulated 10 times. The reproductive ratio and generation period were estimated over 6,655 and 6,702 runs on average for local and global sensitivity respectively, as detailed in Appendix D.

Randomization

Software randomization of agents compliant with different intervention policies, using embedded C++ random number generators. A sample population is generated stochastically.

Blinding

Blinding is not relevant for the simulation analysis.

Reporting for specific materials, systems and methods

We require information from authors about some types of materials, experimental systems and methods used in many studies. Here, indicate whether each material, system or method listed is relevant to your study. If you are not sure if a list item applies to your research, read the appropriate section before selecting a response.

Materials & experimental systems

n/a	Involved in the study	
☒	☐	Antibodies
☒	☐	Eukaryotic cell lines
☒	☐	Palaeontology
☒	☐	Animals and other organisms
☒	☐	Human research participants
☒	☐	Clinical data

Methods

n/a	Involved in the study	
☒	☐	ChIP-seq
☒	☐	Flow cytometry
☒	☐	MRI-based neuroimaging
